# Does pyogenic liver abscess increase the risk of delayed-onset primary liver cancer? Evidence from a nationwide cohort study

**DOI:** 10.1097/MD.0000000000007785

**Published:** 2017-08-25

**Authors:** Chia-Sheng Chu, Che-Chen Lin, Cheng-Yuan Peng, Po-Heng Chuang, Wen-Pang Su, Shih-Wei Lai, Hsuan-Ju Chen, Chi-Jung Chung, Hsueh-Chou Lai

**Affiliations:** a School of Chinese Medicine, China Medical University; b Division of Hepatogastroenterology, Department of Internal Medicine; c Management Office for Health Data, China Medical University Hospital; d School of Medicine, China Medical University; e Department of Family Medicine; f Department of Medical Research, China Medical University Hospital; g Department of Health Risk Management, China Medical University, Taichung, Taiwan.

**Keywords:** biliary tract cancer, hepatoma, infection, pyogenic liver abscess, risk factor

## Abstract

Delayed-onset primary liver cancer (PLC) including hepatocellular carcinoma (HCC) and intrahepatic cholangiocarcinoma (ICC) in patients with pyogenic liver abscess (PLA) is not common. The relationship between PLA and delayed-onset PLC is unclear. We investigated the association in a nationwide cohort study.

From Taiwan National Health Insurance claims data, a cohort of 17,531 patients with PLA was generated after excluding patients with a history of cancer (n = 2034) and those diagnosed with PLC (n = 572) and other cancers (n = 627) within 1 year of a diagnosis of PLA. An age-, sex-, index year-, and diabetes mellitus (DM)-matched control cohort of 70,124 persons without PLA was selected from the same dataset. Both cohorts were followed up until the end of 2011. The risk of PLC was estimated for both cohorts.

The incidence of PLC was nearly 2-fold greater in the PLA group than in the control cohort (29.3 per 10,000 person-years vs. 16.2 per 10,000 person-years). The incidences of HCC and ICC were 1.5- (22.1 per 10,000 person-years vs. 15.0 per 10,000 person-years) and 11-fold greater (6.73 per 10,000 person-years vs. 0.62 per 10,000 person-years), respectively, in the PLA group than in the control cohort. The PLA cohort also had high risks of PLC (adjusted hazard ratio [aHR] = 1.56; 95% confidence interval [CI] = 1.35–1.81), HCC (aHR = 1.34; 95% CI = 1.15–1.57), and ICC (aHR = 6.94; 95% CI = 4.23–11.57).

In conclusion, in this nationwide cohort study, PLA increased the risk of delayed-onset PLC.

## 1 Introduction

Pyogenic liver abscess (PLA), is a type of liver abscess caused by bacteria, is not an uncommon infectious and life-threatening disease with a high mortality rate of approximately 5% to 6%.^[1,2]^*Klebsiella pneumoniae* (*K pneumoniae*) and *Escherichia coli* (*E coli*) were the most common microorganisms among patients with PLA.^[1]^ A high annual incidence of 17.6 per 100,000 people is noted in Taiwan.^[3]^ In the Denmark, the incidence of PLA increased from 0.6 to 1.8 per 100,000 for men and from 0.8 to 1.2 per 100,000 for women between 1977 and 2002.^[2]^ The major comorbidities of PLA are diabetes mellitus.^[1,4]^ intra-abdominal infection, and pancreatic and biliary tract diseases including cholangitis, cholecystitis, diverticulitis, appendicitis, and peritonitis.^[5–7]^

The main infection routes of PLA are hematologic entry from the portal systemic organs because of mucosal defects or disease that compromises barrier function and ascending infection from the pancreaticobiliary system. Some studies illustrated that gastrointestinal premalignant ^[8]^ or malignant lesions ^[9–12]^ are associated with PLA. Primary liver cancer (PLC) including hepatocellular carcinoma (HCC) and intrahepatic cholangiocarcinoma (ICC) can initially present with PLA or arise within 1 year of a diagnosis of PLA were reported by a large-scale cohort study ^[13]^ and some case series reports.^[14–17]^ The associated risk of PLC was unclear after 1 year in patients with PLA (we defined delayed-onset PLC as that diagnosed >1 year after a diagnosis of PLA) because of the relative small sample size^[18]^ and scarcity of reports.^[19]^

At present, no large-scale population-based study has been conducted to evaluate the association between PLA and delayed-onset PLC including HCC and ICC. The aim of this study was to estimate the risk of delayed-onset PLC in patients with PLA using a nationwide population-based database in Taiwan.

## 2 Materials and methods

### 2.1 Data source

Taiwan National Health Insurance (Taiwan NHI) is a nationwide, single-payer health insurance program that is compulsory for all citizens. Taiwan NHI was established in 1995, and 99% of Taiwan's 23 million citizens were covered in 1998. The Taiwanese government ordered the National Health Research Institutes (NHRI) to construct and manage the National Health Insurance Research Database (NHIRD). The NHIRD handles all of the claims data of Taiwan NHI, including the registry for beneficiaries, ambulatory and inpatient care, prescription records, and other medical services. The NHRI renews the database annually. To protect the confidentiality of the insured subjects, the NHRI removed the original identification number and published the database with an encoded identification number to link each medical service file. This study was approved by the Institutional Review Board of China Medical University in central Taiwan (CMU104-REC2–115).

The disease diagnosis record system in the NHIRD is categorized according to the International Classification of Diseases, Ninth Revision, Clinical Modification (ICD-9-CM). The cancer history of each patient was collected from a catastrophic illness patient registry, a subcomponent of the NHIRD. PLA- and PLC-associated comorbidity data were collected from inpatient files.

### 2.2 Study population

**Figure 1:**
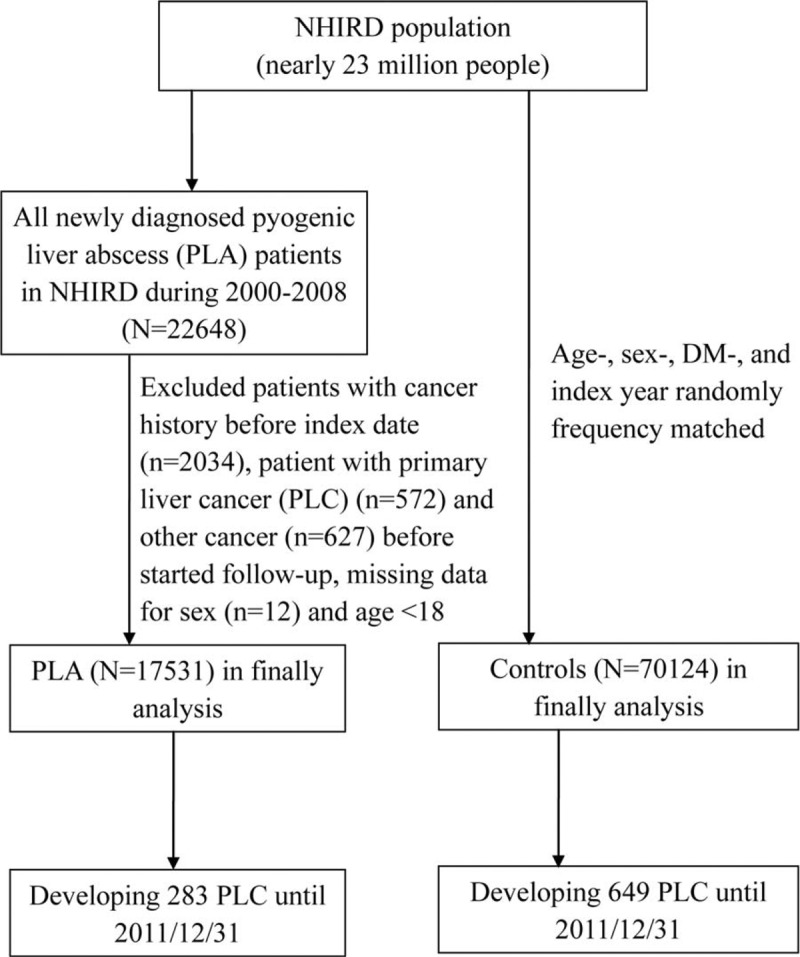
Flow chart of the subject selection process. DM = diabetes mellitus, NHIRD = National Health Insurance Research Database, PLA = pyogenic liver abscess, PLC = primary liver cancer.

We designed a retrospective population-based cohort study to investigate the association between PLA and PLC risk. Figure 1 presents a schematic of the study population selection protocol. The PLA cohort consisted of patients who were newly diagnosed with PLA (ICD-9-CM 572.0) between 2000 and 2008, and the initial date of the PLA diagnosis was set as the index date. The control cohort consisted of individuals with no PLA diagnosis record in the NHIRD, and these individuals were matched by age (per 5 years), sex, and history of DM history (ICD-9-CM 250) with the PLA cohort. The index date of the control cohort was randomly assigned to match that of the matched case. The study cohort was followed up at 1 year after the index date. We excluded subjects younger than 18 years and those with a history of cancer (ICD-9-CM 140–208) before follow-up. The major event of interest in the study was the occurrence of newly diagnosed PLC (ICD-9-CM 155), which could be classified into 3 subtypes: HCC (ICD-9-CM 155.0), ICC (155.1), and others. Follow-up was terminated when an individual withdrew insurance or PLC developed, or on December 31, 2011, whichever occurred first.

We considered PLC-associated comorbidities as confounding factors in this study. PLC-associated comorbidities arising before the index date included hepatitis B virus (HBV) infection (ICD-9-CM 070.2 and 070.3), hepatitis C virus (HCV) infection (ICD-9-CM 070.41, 070.44, 070.51, and 070.54), unspecified chronic hepatitis (ICD-9-CM 070.9, 571.4, 571.8, 571.9), alcoholic liver disease (571.0–571.3), liver cirrhosis (ICD-9-CM 571.5 and 571.6), cholelithiasis (ICD-9-CM 574), cholecystitis (ICD-9-CM 575.0 and 575.1), and cholangitis (ICD-9-CM 576.1).

We also investigated the microorganisms identified concurrently with PLC in patients with PLA. The microorganisms included *Staphylococcus* (ICD-9-CM 038.0 and 041.0X), *E coli* (ICD-9-CM 038.42 and 041.4), *Streptococcus* (ICD-9-CM 038.0 and 041.0X), *Pneumococcus* (ICD-9-CM 038.1X and 041.1X), *K pneumoniae* (ICD-9-CM 041.3), Proteus (ICD-9-CM 041.6), Gram-negative bacteria (ICD-9-CM 038.40, 038.49, and 041.85), and other/unspecified bacteria (ICD-9-CM 038.8, 038.9, and 041).

### 2.3 Statistical analysis

The mean and standard deviation (SD) for age as well as the number and percentage for sex and PLC-associated comorbidities of the study cohort were showed. To assess differences in distribution between the PLA and control cohorts, we used the *t* test for age and the *χ*^2^ test for category variables. The incidence density of PLC was calculated as the number of PLC events divided by the total sum of the follow-up years for each study cohort and presented as the rate per 10,000 person-years. We used the Kaplan-Meier method to estimate the cumulative incidence curves and tested the difference of the curves using the log rank test. Univariate and multivariate Cox proportional hazard regression models were used to estimate the hazard ratios (HRs) and 95% confidence intervals (CIs) for the risk of PLC. We also estimated the association between an increasing frequency of PLA diagnosis and PLC risk and tested the trend for increasing PLC risk with the frequency of PLA diagnosis as a continuous variable using a Cox proportional hazard regression model. Finally, we performed a stratified analysis to estimate the effect of PLA on PLC risk for different demographics and comorbidities.

All data management and analyses were performed using SAS software (version 9.4 for Windows; SAS Institute, Inc., Cary, NC). The cumulative incidence curves were also drawn using SAS software. The level of statistical significance was 2-sided and set at 0.05.

## 3 Results

The PLA and control cohorts consisted of 17,531 and 70,124 people, respectively (Table 1). Because the cohorts were matched for age, sex, and history of DM, the mean age (58 years), sex distribution (male: 62.6%), and history of DM (34.5%) were similar between the cohorts. The frequencies of comorbidities were greater in the PLA cohort than in the control cohort (*P *<* *.0001).

**Table 1:**
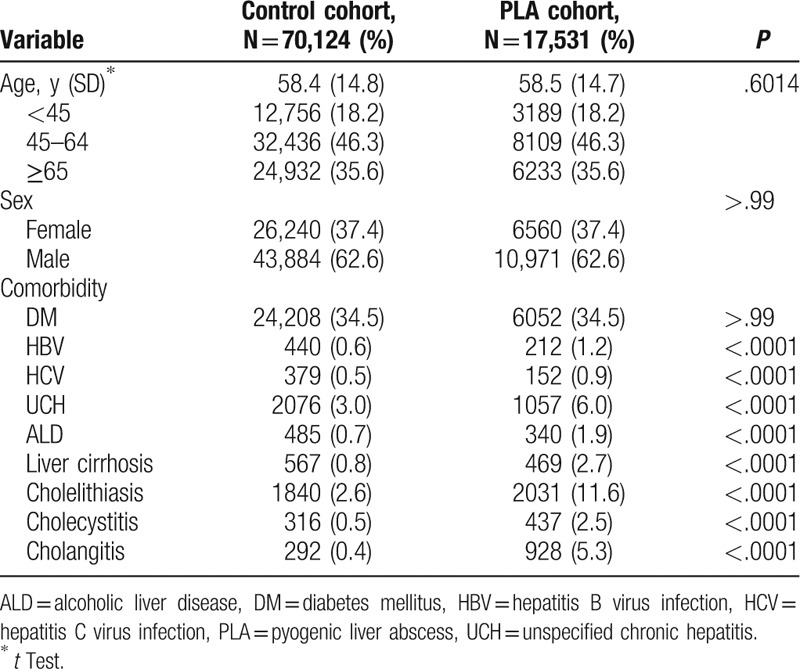
Baseline demographics and comorbidities of the control and PLA cohorts.

**Figure 2:**
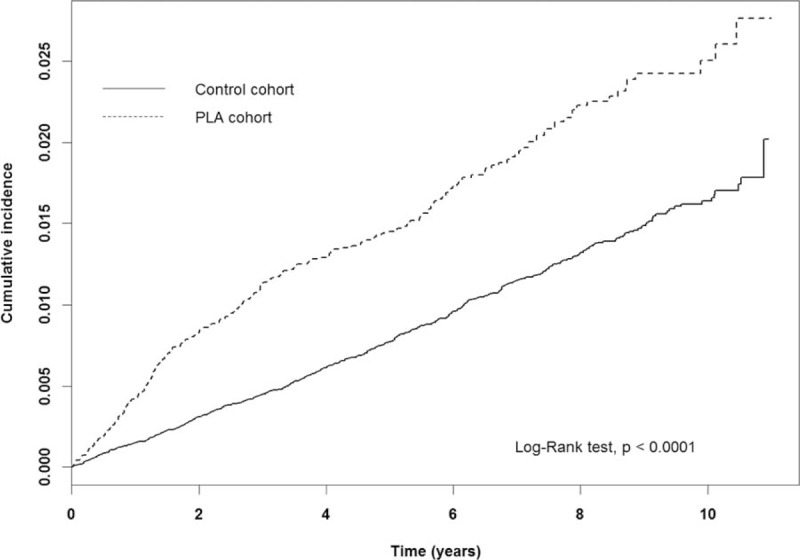
The cumulative incidence curves of primary liver cancer for the pyogenic liver abscess (PLA) and control cohorts.

The incidence of PLC was 29.32 per 10,000 person-years in the PLA cohort and 16.19 per 10,000 person-years in the control cohort (Table 2). Figure 2 presents the PLC cumulative incidence curves for the 2 cohorts. The incidence curve for the PLA cohort was significantly larger than that for the control cohort (*P* for log rank test <.0001). The incidences of HCC and ICC in the PLA cohort were nearly 1.4- and 10-fold higher, respectively, than those in the control cohort. The incidence of the other subtypes of PLC was not different between the cohorts. After adjusting for age, sex, and all comorbidities, PLA was significantly associated with an increased risk of PLC (adjusted HR [aHR] = 1.56; 95% CI = 1.35–1.81). Compared with the control cohort, the PLA cohort had a 1.34- and 6.94-fold greater risk of HCC (aHR = 1.34; 95% CI = 1.15–1.57) and ICC (aHR = 6.94; 95% CI = 4.23–11.38), respectively.

**Table 2:**
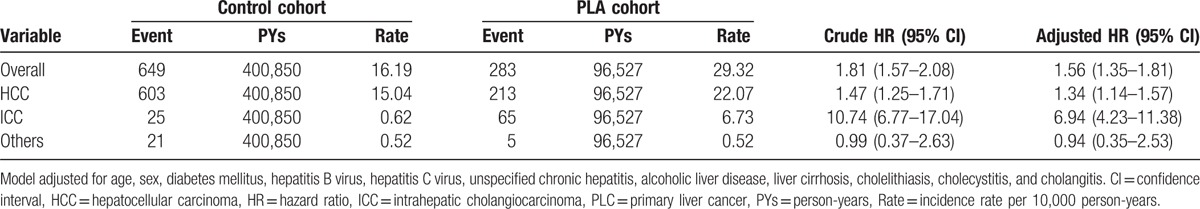
Incidence of PLC and multivariate Cox proportional hazards regression analysis for the study cohort.

Table 3 illustrates the association between an increasing frequency of PLA diagnosis and PLC risk. The incidence of PLC was 16.19 per 10,000 person-years for the control cohort, versus 26.44, 38.54, and 83.56 per 10,000 person-years for <2, 2 to 3, ≥4 diagnoses of PLA, respectively. After adjusting for age, sex, and all comorbidities, compared with the control cohort, patients with <2, 2 to 3, and ≥4 PLA diagnoses had a 1.45- (95% CI = 1.23–1.70), 1.92- (95% CI = 1.49–2.48), and 3.65-fold (95% CI = 1.86–7.13) increased risk of PLC, respectively. The risk of PLC was significantly increased with an increased frequency of PLA diagnosis (*P* for trend <.0001).

**Table 3:**
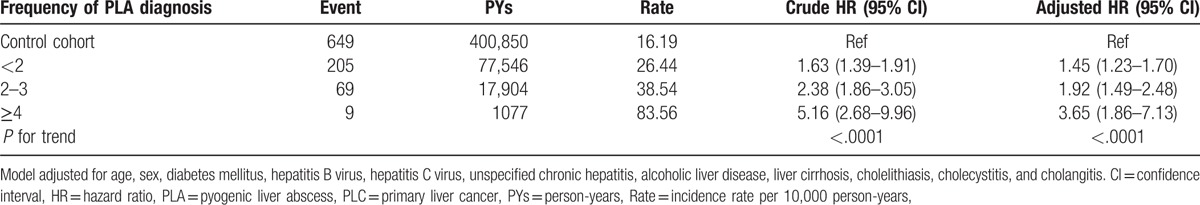
Incidence of PLC and hazard ratios for PLC risk in the control and PLA cohorts.

Table 4 presents the age-, sex-, and comorbidity-specific stratified analyses. Relative to the control cohort, PLA was significantly associated with an increased risk of PLC in patients aged 40 to 59 years and ≥65 years. The PLA cohort had a significantly higher risk of PLC than the control cohort for both males and females. The results also revealed that PLA was only significantly associated with an increased risk of PLC in the study population in the absence of each comorbidity.

**Table 4:**
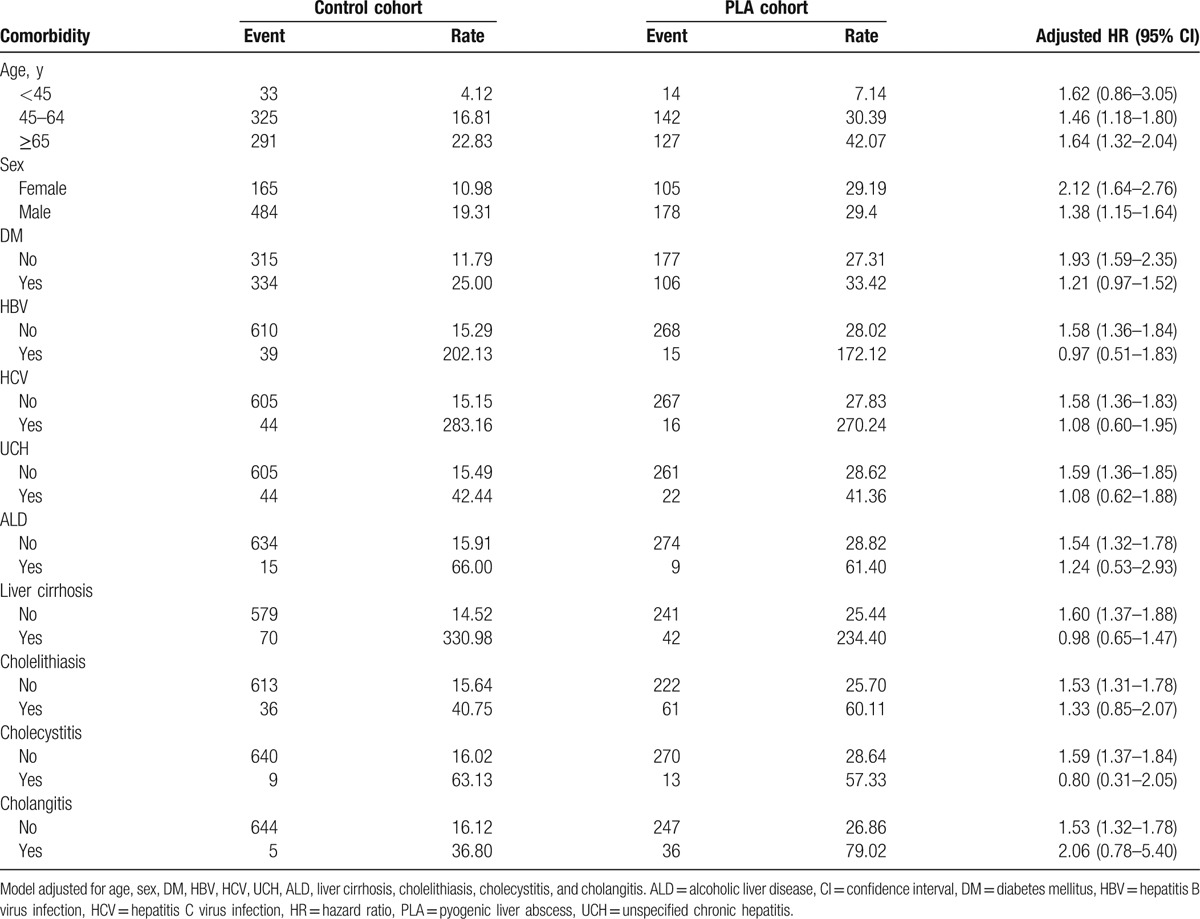
Age-, sex-, and comorbidity-stratified analysis of the risk of PLC in the control and PLA cohorts.

In total, 81.6% of patients with PLA and PLC had positive microorganism test results during the observation period (Table 5). *K pneumoniae* was the major microorganism in patients with HCC (25.5%), and *E coli* was the main microorganism in patients with ICC (34.8%).

**Table 5:**
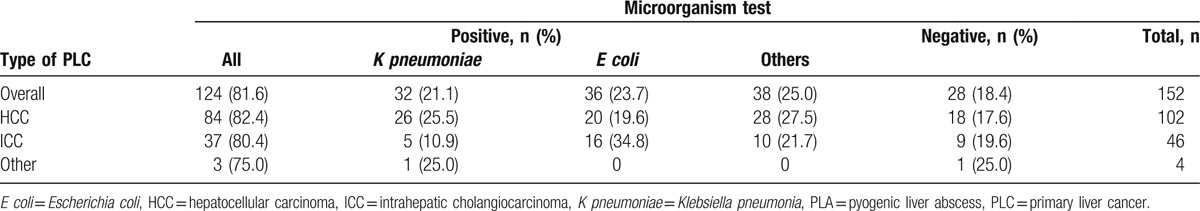
Microorganisms detected concurrently with PLC in patients with PLA (N = 283).

## 4 Discussion

This large-scale, nationwide cohort study is the first to reveal the significantly higher risk of delayed-onset PLC (aHR = 1.56; 95% CI = 1.35–1.81) in patients with PLA, including HCC (aHR = 1.34; 95% CI = 11.14–1.57) and ICC (aHR = 6.94; 95% CI = 14.23–11.38). The incidence of delayed-onset PLC was increased in both sexes in the PLA cohort compared with the control cohort. The risk of delayed-onset PLC was significantly higher in patients with repeated hospitalization because of PLA than in patients with a single PLA-related hospitalization and the controls. *E coli* and *K pneumoniae* were the most common microorganisms detected in patients with PLA and delayed-onset PLC. *K pneumoniae* was the major microorganism in patients with HCC, whereas *E coli* was the most common microorganism in patients with ICC.

HCC and ICC are the 2 most common PLCs, representing 90% and 8%, respectively, of all PLCs.^[20]^ HCC is the fifth most common cancer ^[21]^ and the second leading cause of cancer-related death globally.^[22]^ Meanwhile, 10% of cholangiocarcinomas are ICC.^[23]^ ICC is highly prevalent in Asian (1.3 per 100,000) and Hispanic people (1.2 per 100,000),^[24]^ and its incidence and associated mortality are increasing in several Western countries.^[25,26]^ The dominant risk factors of HCC are cirrhosis, chronic hepatitis B, chronic hepatitis C, alcoholic consumption, obesity, and diabetes,^[27]^ and the major risks of ICC are similar as those of HCC ^[28]^ and include hepatolithiasis.^[29]^

HCC and ICC with PLA as the initial presentation are uncommon, and they are associated with poor outcomes.^[14–17]^ Two major acceptable mechanisms of this presentation are bacterial colonization after spontaneous necrosis in the tumor parenchyma and biliary obstruction with tumor thrombi.^[15]^ Yeh et al^[15]^ and Okuda et al ^[16]^ reported 5 and 10 cases of PLA, respectively, as the initial manifestation of HCC, with mean survival rates of 2.35 and 3.5 months, respectively. Li et al^[17]^ described 5 cases of HCCs and 4 cases of ICC presenting with PLA; nevertheless, the survival time ranged from 8 days to 7.5 months in the ICC group. Chong et al^[30]^ reported 2 cases of HCC and 1 case of ICC that developed as late as 1 year after a diagnosis of PLA (which we defined as delayed-onset PLC). Huang et al^[18]^ did not identify an increased risk of delayed-onset PLC and revealed as a warning sign for PLC in a related small PLA population. Kao et al^[19]^ reported that patients with PLA have a standardized incidence ratio of 2.02 (95% CI = 1.08–3.46) for delayed-onset PLC compared to the general population, However, only 13 cases of delayed-onset PLC were reported, which is an insufficient sample size to distinguish HCC from ICC. In addition, no significantly increased risk of delayed-onset PLC was identified in females with PLA, and thus, the relationship between delayed-onset PLC and PLA remains unclear. This study revealed an increased risk of delayed-onset PLC including HCC and ICC in both sexes after a diagnosis of PLA.

We considered the possibility of different mechanisms between delayed-onset PLC in patients with PLA and those with PLA as an initial presentation of PLC. Inflammation can induce malignant changes in cells.^[31]^ In a study of 102 patients with PLA, KC et al^[32]^ reported ultrasonic evidence of PLA resolution within 2 months after treatment in most patients; however, 8 patients had delay healing with residual abscess after 2 years of follow-up, and 4 patients had calcified lesions after the abscess was resolved. Inflammatory pseudotumors can arise after the onset of PLA,^[33,34]^ and they may be associated with the transformation of PLC.^[30]^ Some literature also revealed an increased risk of lung cancer after pulmonary tuberculosis via a reasonable mechanism of an inflammatory process or scarring.^[35,36]^ This study observed an increased risk of delayed-onset PLC after repeated hospitalization because of PLA.

In patients with PLA, 60% of the detected infectious pathogens are *K pueumoniae*.^[1]^ This study found that *E coli* was the most common pathogen in patients with PLA and delayed-onset ICC. This finding was similar with those of Chen et al^[37]^ and Chuang et al^[38]^ who found that *E coli* and PLA were likely to be associated with a biliary tract disease or hepatobiliary malignancy, whereas a previous study identified *K pneumoniae* as the dominant bacterium in patients with gastrointestinal cancer and PLA.^[11]^

Our results showed the risk of ICC is much higher than HCC in the PLA group. In our previous study found patient with biliary tract infection have an increased risk of digestive system cancers, particularly biliary tract cancer, with hazard ratio 24.45.^[39]^ Up to 20% of pyogenic liver abscess develop from an infected or inflamed biliary tract.^[4]^ The possible mechanism may be the inflammation of biliary tract epithelium is more intensive than hepatocyte.

The use of a large-scale, representative, nationwide, population-based sample to evaluate the risk of delayed-onset PLC in patients with PLA improved the availability of data and the validity of the findings. The large sample size allowed us to perform a stratified analysis to observe comorbidities in patients with PLA. However, this study has several limitations. First, detailed information associated with the risk of PLC, including data on family history of PLC, smoking, alcohol consumption, coffee consumption, high-fat diet intake, physical activity, and body mass index, were not available. Second, data for microorganism infection may have been incompletely coded, thus compromising the identified risk of PLC in patients with PLA.

In conclusion, this population-based study revealed an increased risk and incidence of delayed-onset PLC including HCC and ICC in patients with PLA. Long-term follow-up is suggested for patients with PLA, especially among those with repeated hospitalization because of PLA and slowly healing abscesses.

## References

[1] Chen W Chen CH Chiu KL Clinical outcome and prognostic factors of patients with pyogenic liver abscess requiring intensive care. Crit Care Med 2008;36:1184–8. 18379245 10.1097/CCM.0b013e31816a0a06

[2] Jepsen P Vilstrup H Schønheyder HC A nationwide study of the incidence and 30-day mortality rate of pyogenic liver abscess in Denmark, 1977–2002. Aliment Pharmacol Ther 2005;21:1185–8. 15882238 10.1111/j.1365-2036.2005.02487.x

[3] Tsai FC Huang YT Chang LY Pyogenic liver abscess as endemic disease, Taiwan. Emerg Infect Dis 2008;14:1592–600. 18826824 10.3201/eid1410.071254 PMC2609891

[4] Thomsen RW Jepsen P Sorensen HT Diabetes mellitus and pyogenic liver abscess: risk and prognosis. Clin infect Dis 2007;44:1194–210. 17407038 10.1086/513201

[5] Cohen JL Martin FM Rossi RL Liver abscess. The need for complete gastrointestinal evaluation. Arch Surg 1989;124:561–4. 2712697 10.1001/archsurg.1989.01410050051009

[6] Sherman JD Robbin SL Changing trends in caustics of hepatic abscess. Am J Med 1960;28:943–50. 14445885 10.1016/0002-9343(60)90203-5

[7] Ledeman ER Crum NF Pyogenic liver abscess with a focus on Klebsiella pneumoniae as a primary pathogen: an emerging disease with unique clinical characteristics. Am J Gastroenterol 2005;100:322–31. 15667489 10.1111/j.1572-0241.2005.40310.x

[8] Lai HC Chan CY Peng CY Pyogenic liver abscess associated with large colonic tubulovillous adenoma. World J Gastroenterol 2006;12:990–2. 16521236 10.3748/wjg.v12.i6.990 PMC4066173

[9] Lai HC Chou JW Peng CY Do diabetes patients with Klebsiella pneumoniae liver abscess need further evaluation of the colon for presence of neoplasm in Taiwan. Gut 2009;58 (suppl II):A 145 Abstract.19091833

[10] Lai HC Lin HC Cryptogenic pyogenic liver abscess as a sign of colorectal cancer: a population-based 5-year follow-up study. Liver Int 2010;30:1387–93. 20731775 10.1111/j.1478-3231.2010.02327.x

[11] Lai HC Lin CC Cheng KS Increased incidence of gastrointestinal cancers among patients with pyogenic liver abscess: a population-based cohort study. Gastroenterology 2014;146:129–37. 24095786 10.1053/j.gastro.2013.09.058

[12] Jeong SW Jang JY Lee TH Cryptogenic pyogenic liver abscess as the herald of colon cancer. J Gastroenterol Hepatol 2012;27:248–55. 21777280 10.1111/j.1440-1746.2011.06851.x

[13] Lin YT Liu CJ Chen TJ Pyogenic liver abscess as the initial manifestation of underlying hepatocellular carcinoma. Am J Med 2011;124:1158–64. 22114829 10.1016/j.amjmed.2011.08.012

[14] Huang CI Wang LY Yeh ML Hepatocellular carcinoma associated with liver abscess. Kaohsiung J Med Sci 2009;25:537–43. 19767259 10.1016/S1607-551X(09)70546-7 PMC11917539

[15] Yeh TS Jan YY Jeng LB Hepatocellular carcinoma presenting as pyogenic liver abscess: characteristics, diagnosis, and management. Clin Infect Dis 1998;26:1224–6. 9597257 10.1086/520290

[16] Okuda K Kondo Y Nakano M Hepatocellular carcinoma presenting with pyrexia and leukocytosis: report of five cases. Hepatology 1991;13:695–700. 1849115

[17] Li C Li G Miao R Primary liver cancer presenting as pyogenic liver abscess: characteristics, diagnosis,;1; and management. J Surg Oncol 2012;105:687–91. 21952992 10.1002/jso.22103

[18] Huang WK Lin YC Chiou MJ Pyogenic liver abscess as a warning sign for primary liver cancer: a nationwide population-based study. Asian Pac J Cancer Prev 2013;14:4727–31. 24083734 10.7314/apjcp.2013.14.8.4727

[19] Kao WY Hwang CY Chang YT Cancer risk in patients with pyogenic liver abscess: a nationwide cohort study. Aliment Pharmacol Ther 2012;36:467–76. 22779737 10.1111/j.1365-2036.2012.05212.x

[20] Cong WM Dong H Tan L Surgicopathological classification of hepatic space-occupying lesions: a single-center experience with literature review. World J Gastroenterol 2011;17:2372–8. 21633636 10.3748/wjg.v17.i19.2372 PMC3103789

[21] El-Serag HB Hepatocellular carcinoma: an epidemiologic view. J Clin Gastroenterol 2002;35 (suppl):S72–8. 12394209 10.1097/00004836-200211002-00002

[22] World Health Organization. Mortality Database. WHO Statistical Information System. 2008 Available at: http://www.who.int/whosis. Accessed June 21, 2015.

[23] DeOliveira ML Cunningham SC Cameron JL Cholangiocarcinoma: thirty-one-year experience with 564 patients at a single institution. Ann Surg 2007;245:755–62. 17457168 10.1097/01.sla.0000251366.62632.d3 PMC1877058

[24] McLean L Patel T Racial and ethnic variations in the epidemiology of intrahepatic cholangiocarcinoma in the United States. Liver Int 2006;26:1047–53. 17032404 10.1111/j.1478-3231.2006.01350.x

[25] Khan SA Emadossadaty S Ladep NG Rising trends in cholangiocarcinoma: is the ICD classification system misleading us? J Hepatol 2012;56:848–54. 22173164 10.1016/j.jhep.2011.11.015

[26] Tyson GL El-Serag HB Risk factors for cholangiocarcinoma. Hepatology 2011;54:173–84. 21488076 10.1002/hep.24351 PMC3125451

[27] Mittal S El-Serag HB Epidemiology of hepatocellular carcinoma: consider the population. J Clin Gastroenterol 2013;47:S2–6. 23632345 10.1097/MCG.0b013e3182872f29 PMC3683119

[28] Palmer WC Patel T Are common factors involved in the pathogenesis of primary liver cancers? A meta-analysis of risk factors for intrahepatic cholangiocarcinoma. J Hepatol 2012;57:69–76. 22420979 10.1016/j.jhep.2012.02.022 PMC3804834

[29] Zhou YM Yin ZF Yang JM Risk factors for intrahepatic cholangiocarcinoma: a case-control study in China. World J Gastroenterol 2008;14:632–5. 18203300 10.3748/wjg.14.632 PMC2681159

[30] Chong VH Lim KS Pyogenic liver abscess as the first manifestation of hepatobiliary malignancy. Hepatobiliary Pancreat Dis Int 2009;8:547–50. 19822502

[31] Grivennikov SI Greten FR Karin M Immunity, inflammation, and cancer. Cell 2010;140:883–99. 20303878 10.1016/j.cell.2010.01.025 PMC2866629

[32] KC S Sharma D Long-term follow-up of pyogenic liver abscess by ultrasound. Eur J Radiol 2010;74:195–8. 19217231 10.1016/j.ejrad.2009.01.017

[33] Tsou YK Lin CJ Liu NJ Inflammatory pseudotumor of the liver: report of eight cases, including three unusual cases, and a literature review. J Gastroenterol Hepatol 2007;22:2143–7. 18031372 10.1111/j.1440-1746.2006.04514.x

[34] Horiuchi R Uchida T Kojima T Inflammatory pseudotumor of the liver. Clinicopathologic study and review of the literature. Cancer 1990;65:1583–90. 2155699 10.1002/1097-0142(19900401)65:7<1583::aid-cncr2820650722>3.0.co;2-l

[35] Engels EA Shen M Chapman RS Tuberculosis and subsequent risk of lung cancer in Xuanwei, China. Int J Cancer 2009;124:1183–7. 19058197 10.1002/ijc.24042 PMC2610239

[36] Yu YH Liao CC Hsu WH Increased lung cancer risk among patients with pulmonary tuberculosis: a population cohort study. J Thorac Oncol 2011;6:32–7. 21150470 10.1097/JTO.0b013e3181fb4fcc

[37] Chen SC Wu WY Yeh CH Comparison of Escherichia coli and Klebsiella pneumoniae liver abscesses. Am J Med Sci 2007;334:97–105. 17700198 10.1097/MAJ.0b013e31812f59c7

[38] Chuang HC Chen TL Chiang DH Clinical and bacteriological characteristics of pyogenic liver abscess in non-diabetic patients. J Microbiol Immunol Infect 2009;42:385–92. 20182667

[39] Tsai TY Lin CC Peng CY The association between biliary tract inflammation and risk of digestive system cancers. Medicine 2016;95:31 (e4427). 10.1097/MD.0000000000004427 PMC4979819 27495065

